# The Combination of Metals for Filling Teeth

**Published:** 1888-05

**Authors:** C. T. Tockwell

**Affiliations:** Springfield, Mass.


ARTICLE II.

### THE COMBINATION OF METALS FOR FILLING TEETH.

BY DR. C. T. TOCKWELL, SPRINGFIELD, MASS.

[Read before the New York Odontological Society, October 3, 1887.]

For eight or ten years past I have been endeavoring, by experiment and observation, to determine the value, or otherwise, of a combination of metals over a single metal as material for filling teeth. As a result, I have arrived at certain well-defined convictions. But in considering the matter with reference to a response to your invitation to write upon the subject, for this occasion, I am reminded of a prominent author's exclamation when shown that others had long since given voice to substantially his own thoughts with reference to certain matters. It will not say with this author, "Confound those ancients who are always stealing my ideas," but rather that I am happy—on this occasion at least—in the fact that the experience and observation of so many may be cited as agreeing in the opinion that a combination of metals is of much greater value, in many cases, than any one of the various metals used alone.

As far as I am informed, there is a universal agreement, on the part of those who have given the matter any extended experimental attention, in regard to the *saving* qualities of certain combinations of metals. Personally, I do not hesitate to assert that the employment of a combination of metals as a filling-material is eminently successful as a means of prolonging the usefulness of a very large class of teeth. However, much may be claimed in favor of gold, or any other metal, I am fully convinced that every operator is often called upon to deal with cases where a combination of metals is far preferable from the stand-point of *comfort* to be derived and the *durability* of operations.

Tin and gold, for instance, combined in the same cavity, express a preservative power that either, used alone, fails to exhibit.

This is a matter of such universal acknowledgment, and doubtless one so generally acted upon by those present, that I shall not affront your intelligence or courtesy by occupying any time in the attempt to demonstrate the fact. A benefit may, however, be derivable from a comparison of individual methods of practice, and by a study of the relations that exist between the resulting phenomena—when a combination of metals is used—and the cause, or rather the antecedents, of such phenomena.

I will dwell briefly upon these two points:

First, as to the general methods which, in my own practice, have been found to be most satisfactory, together with the resulting phenomena observed.

For some years past, the combinations that I have used consist entirely of amalgam and gold, and "Robinson's fibrous and textile metallic filling" and gold. The latter, for the sake of brevity, I shall hereafter designate by the term "Robinson's foil." When employing the above combinations they are, of course, used in the order named. In the majority of cases I adopt the amalgam and gold combination; but of late, as the result of observation, I am using more extensively the Robinson foil and gold combination. This is so, partly, because of the fact that by this combination operations can be completed at a single sitting; but more especially because of the most excellent results observed.

Robinson's foil is found valuable in starting large crown fillings that are to be finished with gold. But the cases where it serves the best purpose are in starting fillings on the approximal surfaces of teeth, especially of the bicuspids and molars, when not too badly broken down. Enough of it should be used to form, when finished, an external border of this metal at the cervical margins of the cavity, else the good effect will not be so apparent. If, however, the tooth is badly broken down, or it is of a soft, frail texture, my usual method is to start the filling with amalgam, and at a subsequent sitting finish with gold. My general practice is to restore the normal contour of the tooth with amalgam, at the first sitting, and subsequently cut away such portions of the amalgam as may be deemed desirable, replacing the same with gold, so that when finished, especially with the anterior teeth, nothing but gold shall appear to ordinary observation. This is not a difficult task in any save the most extreme cases. Good anchorage may be secured in and around the amalgam, and the gold can be so placed as to conceal from view all traces of the amalgam. A sort of chemical union takes place between the gold and amalgam, so that from a comparatively slight point of anchorage the gold may be built up and carried well over a slightly-rounded edge of amalgam, thus hiding it from view. The amalgam will become dark in shade, and perhaps black, but the gold retains its native appearance.

In almost any case of badly broken-down teeth the original contour of the crown may be easily restored with amalgam, even if the natural crown is almost or quite gone, and when once hardened it can as easily be faced with gold. In the case of incisors and cuspids, and often the bicuspids also, the amalgam should be cut away along the entire anterior surface, in order to avoid discoloration of that portion of the remaining tooth. In rare cases I find it advisable first to fill the anterior surface with gold, and finish by the use of amalgam in that part of the cavity which presents itself on the lingual surface.

By the use of amalgam and gold many cases are successfully treated that otherwise would have to be "crowned;" and I believe that it affords, in effect, a more substantial and permanent crown than would result from any method with which I am acquainted. I have restored several crowns of superior cuspids in this manner which have done excellent service for six or more years, and which seem to-day as promising as when the work was done.

So much as regards methods. I need hardly remind you that I am not advocating this practice without discrimination as regards the class of teeth which come into our hands for treatment. Where the teeth are hard, strong and well-conditioned, gold is good enough and will serve an excellent purpose. But when we have to deal with that large and utterly disheartening class denominated "soft" teeth, manifesting that treacherous, rapid character of disease called "white decay," accompanied by characteristic environments and constitutional tendencies, we stand before a condition of affairs that challenges a broader and wider treatment than lies within the resources of a mere gold hobbyist. The various cements and plastics may be and are used extensively; but the necessity of frequent renewals is discouraging to patient and operator alike. Whereas with the method I have described, among the several and important advantages that I will notice are:

First, the relative permanency of such work. I have never yet had to remove, replace, or patch a single case.

Second, it affords an economy of time, labor, and consequently of expense, to the patient.

Third, and more important, cavities may be filled in this way and no disagreeable and dangerous thermal effects follow, which, if filled with gold or amalgam alone, would cause the patient much discomfort, to say the least. If a combination of metals as a filling-material possessed no other advantage over any one used alone than that of materially lessening the subsequent thermal influences upon the pulp, it would be sufficient to demand our serious consideration. An entire paper might well be written, taking for its subject this point alone. Just here it is where Robinson's foil is found to be of so much value in starting large crown cavities which are to be finished with gold. I believe its use in crown cavities results in other advantages than that of an avoidance of thermal effects, but this is the one most apparent.

Fourth, and by way of hypothesis, I wish to remark that in all my experience with this method—covering some ten years of time and hundreds if not thousands of cases —I have never had a single case where, to my personal knowledge, a pulp has died under one of these combined metal fillings. Furthermore, I am pretty well convinced that if a pulp should die under these circumstances no putrefaction would occur, and consequently no abscess would follow.

Thermal influences will account for the death of thousands of pulps under gold fillings, and this is the beginning of a series of events which result in abscesses. But there are other effects brought about by the combination of metals than that of the avoidance of thermal influences, and which in my judgment are calculated to aid in the preservation of the life of the pulp, or, in case it does become devitalized, to prevent those chemical changes that result in inflammation and abscess, to which allusion will be made as we proceed.

Fifth, I think it may be said that there invariably follows an improvement tn the physical condition of the teeth when either of the combinations I have named is used; such an improvement as would not follow if either of the various metals was used alone. I might cite hundreds of cases in my own practice in support of this posftion—teeth that were soft, frail, and subject to rapid decay; teeth that had been filled with gold and the whole list of plastic msterials, and still persisted in going to destruction in spite of renewals early and often. Many such cases have come into my hands and are now being "saved" by resorting to the treatment and methods I have described. Cases can be presented where I think it may be safely said that fillings made of a combination of metals have already done good service for more *years* than may be numbered by the *months* of former fillings of gold and the various plastics. These cases of soft, frail teeth, when treated in this way, seem to, and I am convinced do, become stronger and more dense in a comparatively short time.

There is, therefore, more involved in this method of treating a certain class of teeth than the mere *mechanical adaptation* of the materials used and the *mechanical exclusion* of outside deleterious agents, whatever they may be. With all due respect for those who had views to the contrary, I am fully convinced that, as a result of this combination of metals, a therapeutic effect is realized and a physiological stimulus is engendered which will, in a large measure, account for the favorable phenomena observed.

We are brought thus to our second point,—the relation that exists between the resulting phenomena and the antecedents of such phenomena.

On a former occasion, before another society, my views were given at some length relative to this point. Further observation and consideration have failed to change these views to any material extent. I then stated that, in my judgment, the effects above named may all be primarily accounted for upon the theory of *galvanic action* as a basis of causation.

In regard to the question of thermal influences, there is no doubt but that Robinson's foil is a better non-conductor than gold; and this would, in part at least, account for the immediate favorable results. But there can be little doubt that the galvanic action set up by this combination, taken in connection with the fluids of the mouth, lends an impulse toward the removal of those physiological sensations resulting from thermal influences. There can be no doubt at all that the combination of amalgam and gold, when placed in contact with the teeth and fluids of the mouth, will create an electric current. And I am assured by competent authority that, when a tooth is so filled, the current will flow in the following direction, viz., from the amalgam down through the body of the tooth to the pericementum, from it to the saliva, and from it or through it to the gold. Now, if this is true, there follows a very significant fact, and one that should be particularly noted: The cervical border of a cavity so filled is protected by and enveloped in an *electric current*; and this current renders a certain defined territory or space about such margins *thoroughly aseptic*. Micro-organisms cannot live in this current, and such portions of the oral cavity are free from the chemical changes that result from the agency of these organisms. Have we not a very suggestive fact, taken in connection with the prevalent theory relative to the etiology of dental caries? Approximal spaces are the dangerous points. Approximal cavities, filled with gold or other metals used alone, are very liable to prove short-lived, especially if the teeth stand in close apposition. It is to these spaces that we call the attention of our patients, charging them to be especially careful and thorough in cleansing. Why? Is it not that fermentation and consequent chemical action may be avoided? If this be so, you will perceive the significance of an electric current at these points that will effectually prevent fermentation and chemical action. It would thus seem that we have hit upon the relation of these combination fillings to the fact that they require less subsequent patching than our beautiful operations with gold when used alone in such cavities. In other words, one of the generally observed phenomena, in connection with these fillings, is linked to its antecedents. And here, also, in the explanation of or ground for the hypothesis before alluded to, that a dead pulp in a tooth so filled will not undergo that process of chemical change that results in abscess. Abscesses are held to-day to be dependent upon the activities of a certain class of micro-organisms; and this electric current is fatal to these organisms. There is too much *lightning* about the region of the pulp-canals for their safety and comfort.

But in regard to the action of this electric current about the cervical borders of cavities, rendering them less liable to be affected by a renewal of decay, there results, in addition, to the *mere presence* of the current, another antiseptic element, viz., the *product* of this galvanic action. The oxidation of the tin and silver that follows aids in rendering the space about such fillings antiseptic. The influence of this product, also, upon the immediately adjacent dentine is sufficient to render it less liable to be acted upon than is the tissue surrounding a gold filling. The tissues themselves are therefore, to an extent rendered antiseptic.

But, beyond these more direct therapeutic effects resulting from galvanic action, there remains another that should not escape attention. I refer to its physiological effect.

That there should be a *stimulating of physiological* function as a result of this galvanic current, seems to be a reasonable hypothesis, and one that is based upon general physical analogy. All the tissues belong to the same body, are under subjection to the same general laws, and will exhibit analogies under similar conditions and circumstances. Electricity, in various forms, is used to-day more extensively than ever before in the treatment of abnormal conditions. It is used to stimulate physiological action. In many cases of tumors, chronic sores, atrophy, etc., it is employed with gratifying success by many of the most prominent specialists. The well-known method of treating bed-sores might be cited. A plate of pure silver, the size of the sore, is put over the affected part; this plate of silver is then connected, by means of a wire, to a piece of zinc which is allowed to rest upon a healthy part of the body. A slight galvanic current is thus established, which proves successful in the removal of these very troublesome sores. It seems reasonable to suppose, at least, that we have here an analogy with the favorable action of the combination of amalgam and gold, or tin and gold, upon diseased dental tissue. With the healing effect, or something analogous to healing in the soft tissues, and restoration of the parts to normal action, goes the restoration of the sensitive organs; and, in consequence of this, the normal feeling or sensation to heat and cold is restored.

Further than this, my observation is a good deal at fault if there is not an improvement in the density and general condition of the teeth very soon after these fillings are introduced; and I cannot regard it as at all improbable that the resultant galvanic action is the immediate antecedent of such improvement, and of an increased resisting power on the part of the tooth, which of itself is a fact of prime importance.

Now, gentlemen, if these things are true, or even approximately true, it is clear that our patients would be greatly benefitted if a much larger use of a combination of metals were to be generally adopted than is the case to-day. The advantages accruing vastly outweigh any minor objections which need to follow. Of course, this kind of work, as is the case with all others, may be slovenly performed, and the teeth can be made to appear unsightly, to say the least. It is not a method that delegates to the past all skill, care, and the exercise of good judgment. When these are exercised, all objections to its use may be reduced to an insignificant degree.—*Dental Cosmos.*

